# A systematic approach to performing a comprehensive transesophageal echocardiogram. A call to order

**DOI:** 10.1186/1471-2261-9-18

**Published:** 2009-05-13

**Authors:** Avinash A Kothavale, Susan B Yeon, Warren J Manning

**Affiliations:** 1 From the Department of Medicine (Cardiovascular Division), Beth Israel Deaconess Medical Center, Harvard Medical School, Boston, Massachusetts, USA

## Abstract

**Background:**

While the order for a clinical transthoracic examination is fairly standardized, there is considerable variability between laboratories and even among physicians in the same laboratory with regard to the order for transesophageal echocardiograms (TEE). A systematic approach is desirable for more efficient use of physician and patient time, avoidance of inadvertent omission of important views, and to facilitate study review.

**Methods:**

We propose a standardized approach to TEE data acquisition in which cardiac structures are systematically identified and characterized at sequential positions and imaging planes to facilitate organized, efficient and comprehensive assessment.

**Results:**

Our approach to TEE study begins in the mid-esophagus with the imaging plane at 0°. Based on the specific indication for the TEE, a cardiac structure (e.g., mitral valve, left atrial appendage, or interatrial septum) is chosen as the primary focal point for a comprehensive, multiplane analysis. This structure is assessed in 20° – 30° increments as the imaging plane is advanced from 0° to 165°. Using the aortic valve as a reference point, pertinent cardiac structures are then assessed as the imaging plane is reduced to 135°, to 90°, to 40 – 60° and then back to 0°. The probe is then advanced into the stomach to obtain transgastric images at 0°, 90°, and 120°. Finally, the thoracic aorta and pulmonary artery are assessed as the probe is withdrawn from the body. Using this method, an organized and comprehensive TEE can be performed in 10 – 15 minutes.

**Conclusion:**

A standardized and systematic TEE approach is described for efficient and comprehensive TEE study.

## Background

Transesophageal echocardiography (TEE) is a moderately invasive technique used to image the heart and great vessels by placing an ultrasound probe into the patient's esophagus and stomach. Compared to transthoracic echocardiography (TTE), the distance between the ultrasound transducer and the heart is diminished with minimal intervening air or body structures, enabling the use of higher frequency probes that yield improved spatial resolution. TEE technology has evolved significantly since 1974 when the first rigid M-mode device was described by Frazin [1]. Subsequent advancements in TEE technology have led to monoplane, followed by bi-plane, and now smaller, flexible, high frequency multiplane probes capable of two dimensional, M-mode, and Doppler imaging. This remarkable progress has allowed TEE to become a routine imaging modality for the diagnosis and management of a wide range of cardiovascular diseases and performed in the ambulatory, intensive care, and operative room settings. This advancement from monoplane to multiplane imaging has also increased the complexity of the TEE examination. An early description of a systematic multiplane examination for patients under conscious sedation was published by Seward et al. in 1993 [2]. Later, in 2001, Shanewise reported a comprehensive method of performing an intraoperative TEE [3]. These papers as well as current echocardiography textbooks [4,5] and Society recommendations [6,7] are based on sequentially imaging specific anatomical structures, but they provide limited guidance for a specific "order" for a study that would provide a comprehensive assessment of a patient. A common recommendation by all of these authors is to begin the TEE examination by focusing on the structure of primary interest so that the main objective is achieved if the study is terminated prematurely due to hemodynamic instability or other disruption [4-8]. A systematic method of TEE image acquisition is important, just as an orderly approach to a TTE exam (parasternal long axis, followed by parasternal short axis, apical four chamber, apical five chamber, apical two chamber, apical three chamber, subcostal, and suprasternal views) facilitates effective use of sonographer and patient time, avoids inadvertent omission of important views, and also facilitates subsequent review. These issues are especially pertinent for a moderately invasive procedure such as TEE. In this report, we present an example of an organized approach for clinical TEE in which the cardiac structures are systematically identified at specified sequential multiplane transducer angle positions to facilitate organized assessment. Using this method, a comprehensive TEE can be performed in 10 – 15 minutes.

A comprehensive review of the indications and morbidity for TEE is beyond the scope of this paper, and the reader is referred elsewhere for discussions of these topics [9-12]. In preparation for performing a TEE, it is important to review the indication for the study as well as the patient's medical history as this clinical information is crucial to the TEE exam.

## Methods

### A Structured Comprehensive TEE Examination

There are several alternative methods of organizing the acquisition of TEE images. Some operators choose to answer the referral question as quickly as possible and then terminate the study. Others attempt to perform a comprehensive study on all patients referred for TEE. Except for certain emergency situations, it is our practice to initially focus on the primary area of interest, but to perform a comprehensive study on all patients thereby providing an evaluation of all four cardiac chambers, all four valves, the left and right atrial appendages, interatrial septum, coronary ostia, coronary sinus, thoracic aorta, pulmonary artery, vena cavae, and pericardium. An overview is provided in Table 1.

**Table 1:** Summary of TEE Examination

TEE probe distance from incisors	Crystal Orientation	Maneuvers/View	Visualized Anatomy
~35–40 cm (mid esophagus)	0°	Probe Rotation	LV, MV; RA, TV; LVOT, AV, IAS; LA, LAA

	20–30° Incremental to 165°	Crystal rotation about area of interest	e.g., MV, LAA, IAS

	135°	LV long axis	MV, AV, ascending Ao

		Anterior/clockwise probe rotation	TV, RA, RAA, IAS, SVC; RUPV, RLPV

	90°	Two-chamber	MV, LV, LA

		Clockwise/anterior probe rotation	PV, RVOT; TV, RA, RAA; IVC, IAS, SVC; RUPV, RLPV

	40–60°	Short Axis	AV, TV, IAS; LAA

~40 cm (distal esophagus)	0°	Passing through GE junction	RAA, CS, TV

~45 cm (gastric)	0°	LV short axis	LV, RV

	90°	LV two-chamber	LV

	120°	LV long-axis	LV

		Clockwise/anterior rotation	RA, TV, RV, PV

~45–50 cm with graded 2–3 cm withdrawal	0°		Descending Ao

	90°	(if plaque seen)	Descending Ao

		Clockwise/anterior rotation	Aortic arch, PA, PV

Ao = aorta; AV = aortic valve; CS = coronary sinus; GE = gastroesophageal; IAS = interatrial septum; IVC = inferior vena cava; LA = left atrium; LAA = left atrial appendage; LLPV = left lower pulmonary vein; LUPV = left upper pulmonary vein; LV = left ventricle; LVOT = left ventricular outflow tract; MV = mitral valve; PA = pulmonary artery; PV = pulmonic valve; RA = right atrium; RAA = right atrial appendage, RLPV = right lower; RUPV = -right upper pulmonary vein; RVOT = right ventricular outflow tract; SVC = superior vena cava; TV = tricuspid valve pulmonary vein

### Study Overview

Our approach to a comprehensive TEE study begins in the mid-esophagus, approximately 35 centimeters (cm) beyond the patient's incisors with the imaging plane at 0°. Based on the indication for the TEE, a cardiac structure such as the aortic valve, mitral valve, left atrial appendage, or interatrial septum is chosen for initial analysis. This structure is assessed at increasing (20° – 30°) image plane increments as the imaging plane is advanced to 165°. Using the aortic valve as a reference point prior to adjusting transducer crystal angles, pertinent cardiac structures are then assessed as the transducer crystal angle is then reduced to 135°, 90°, 50 – 60° and back to 0°. The probe is then advanced into the stomach, approximately 40 – 50 cm beyond the incisors, to obtain transgastric images at 0°, 90°, and 120°. Finally, the thoracic aorta and pulmonary artery are assessed as the probe is withdrawn from the body.

The TEE probe can be manipulated in multiple ways. The following terms are used here to describe probe manipulation:

1) Advance the probe = push the transducer tip forward towards the stomach

2) Withdraw the probe = pull the probe backward towards the oropharynx

3) Anteflex (flex) = turn the large wheel anteriorly (clockwise)

4) Retroflex (extend) = turn the large wheel posteriorly (counterclockwise)

5) Turn clockwise = turn the control handle clockwise (tip of the probe turns anteriorly)

6) Turn counterclockwise = turn the control handle counterclockwise (tip of the probe turns posteriorly)

## Results

### Study initiation

**Figure 1:**
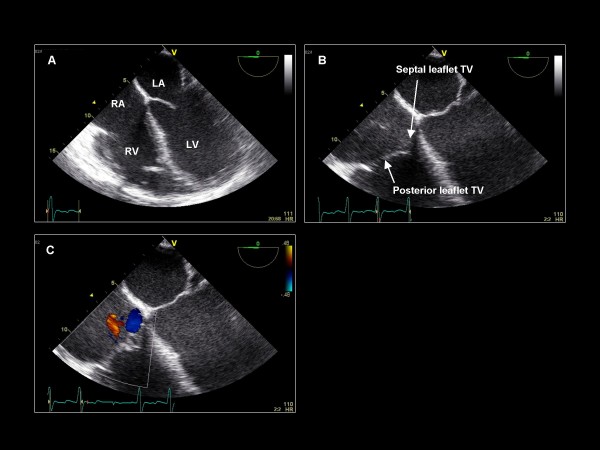
**Imaging plane 0°, (A) The mid-esphageal four chamber view**. (B) The tricuspid valve anatomy with septal and posterior leaflets. (C) Color Doppler applied to the tricuspid valve demonstrating trivial tricuspid regurgitation (orange jet). LA = left atrium, LV = left ventricle, RA = right atrium, RV = right ventricle, TV = tricuspid valve.

**Figure 2:**
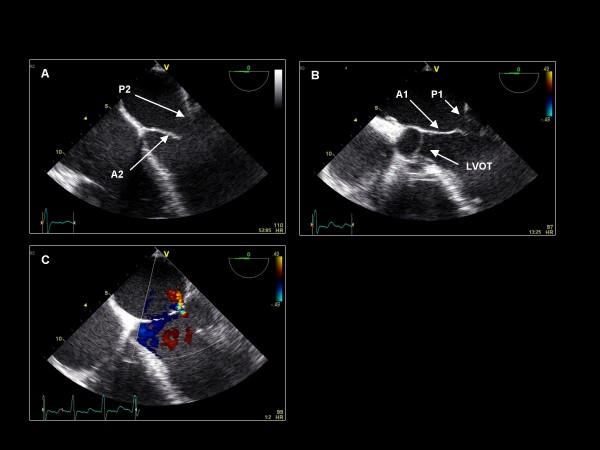
**Imaging plane 0°, (A) The probe is advanced and retroflexed to remove the left ventricular outflow tract (LVOT) from view and demonstrate the A2 and P2 scallops of the mitral valve**. (B) The probe is withdrawn and anteflexed to demonstrate the LVOT and the A1 and P1 scallops of the mitral valve. (C) Color Doppler applied to the mitral valve demonstrating very mild mitral regurgitation.

**Figure 3:**
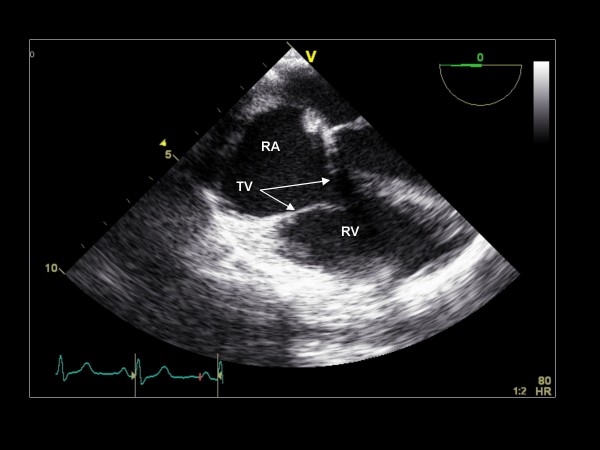
**Clockwise/anterior probe rotation with imaging plane 0° demonstrating the right atrium (RA), tricuspid valve (TV), and right ventricle (RV)**.

**Figure 4:**
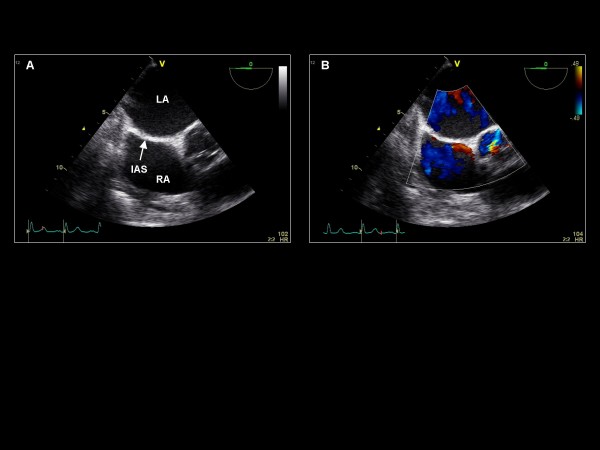
**Further withdrawal and anterior rotation of the probe at imaging plane 0° reveals the (A) The interatrial septum (IAS)**. (B) Color Doppler demonstrates no flow across the IAS.

**Figure 5:**
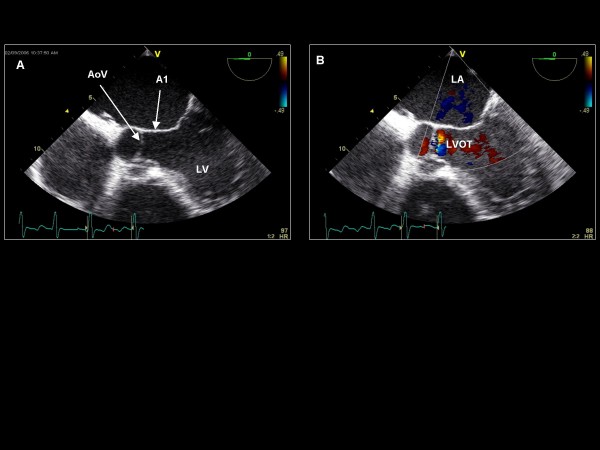
**Imaging plane 0°, (A) The aortic valve (AoV) and A1 segment of the mitral valve**. (B) Color Doppler applied to the aortic valve demonstrating trivial aortic regurgitation within the LVOT.

**Figure 6:**
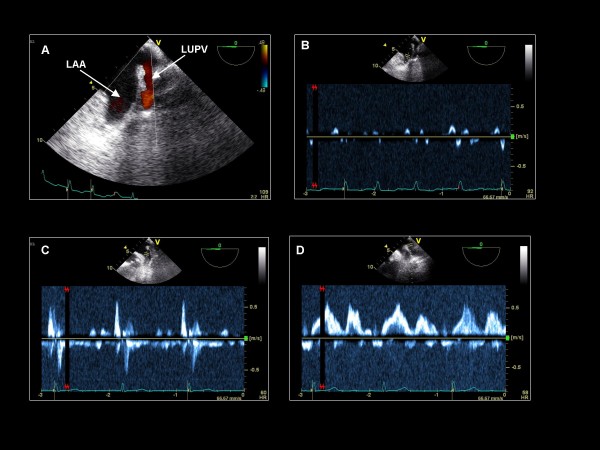
**Imaging plane 0°, (A) The left atrial appendage (LAA) and the left upper pulmonary vein (LUPV)**. Pulse wave Doppler of the LAA demonstrating low emptying velocities in a patient with (B) permanent atrial fibrillation and (C) sinus rhythm. (D) Pulse wave Doppler of the LUPV demonstrating a normal flow pattern.

We begin the TEE examination in the mid-esophagus with the TEE imaging plane at 0° and a four-chamber view, demonstrating the basal and mid segments of the inferoseptum and anterolateral LV walls (Figure 1). This view demonstrates the septal leaflet of the tricuspid valve, and if the probe is retroflexed, the posterior leaflet of the tricuspid valve. Color Doppler and continuous wave (CW) Doppler are applied to assess for regurgitation. If color Doppler suggests an appropriate orientation of the TR jet, CW Doppler is acquired to quantify the RV systolic pressure. With the probe retroflexed and with the probe slightly advanced to exclude the left ventricular outflow tract (LVOT), the A2 scallop and the P2 scallop of the mitral valve are visualized (Figure 2). Flexing and/or withdrawing the probe demonstrate the LVOT as well as the A1 and P1 scallops of the mitral valve. Retroflexing and/or advancing the probe demonstrate a foreshortened ventricle as well as the A3 and P3 scallops [13]. Color Doppler is applied to assess for valvular regurgitation. By advancing the probe and turning the probe clockwise, the right atrium (RA), tricuspid valve (TV), and right ventricle (RV) are visualized (Figure 3). Next, the probe is withdrawn slightly and rotated clockwise to visualize the interatrial septum. Color Doppler is applied to assess for an atrial septal defect (Figure 4). The TEE probe is then rotated counterclockwise and anteflexed in order to demonstrate the LVOT and aortic valve. Color Doppler is applied to assess for aortic regurgitation (Figure 5). Finally, the probe is turned counterclockwise, and anteflexed to visualize the left atrial appendage (LAA) and the left upper pulmonary vein (LUPV) (Figure 6). Pulsed wave Doppler are performed with the sample volume at the mouth of the LAA and LUPV.

### Imaging Plane Advancement 0 – 165 Degrees

**Figure 7:**
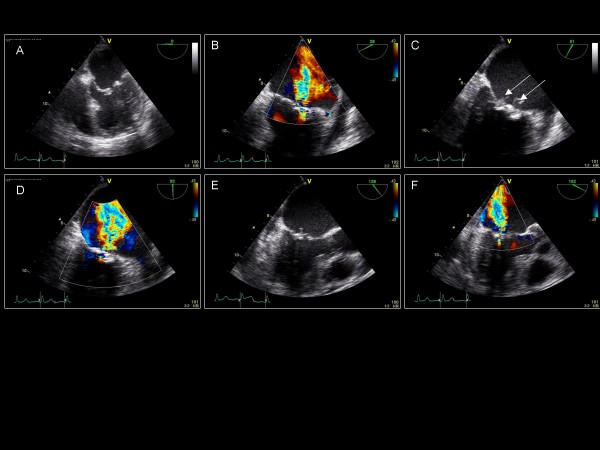
**A-F) Sequential 0° – 165° imaging planes in a patient with mitral valve endocarditis (C: arrows) and moderate to severe mitral regurgitation (B, D, F)**.

A comprehensive, multiplane assessment with a specific cardiac structure as a primary focal point is then performed, based on the indication for the TEE. For example, if the TEE is being performed on a patient in atrial fibrillation to exclude the presence of a LA/LAA thrombus, the LAA is chosen for this next interrogation. In a patient with mitral valve prolapse and suspicion of endocarditis, the mitral valve is chosen and will be presented here in more detail. The probe is turned to bring the patient specific structure of interest into the center of the screen at 0°. Then, the imaging plane is increased in 15–20° increments from 0° to 165°. After each change in imaging plane, 2D and color Doppler images are recorded. If a valve or the interatrial septum is the anatomic structure of choice, color Doppler is applied after each transducer angle change to assess for regurgitation or interatrial flow, respectively. If the interatrial septum has been selected as the anatomy of specific interest, attention is made to the optimal imaging plane for subsequent saline contrast injection. Selected sequential images for a TEE examination of a patient with suspected mitral valve endocarditis is provided in Figure 7.

### 135° Imaging plane

**Figure 8:**
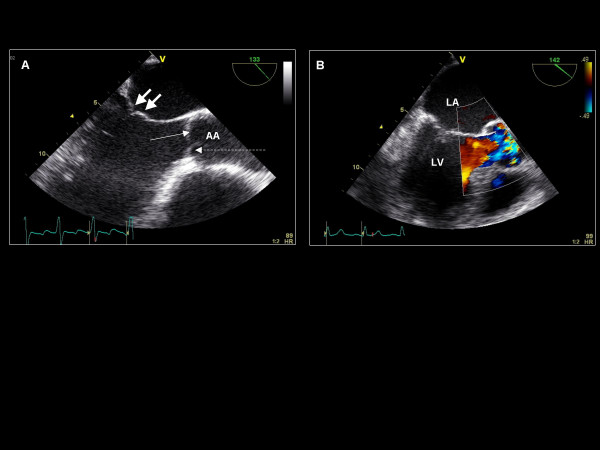
**Imaging plane ~135°, (A) The aortic valve and left ventricle long axis view with the left or non-coronary leaflet (solid thin arrow) and right coronary cusp (dashed arrow) of the aortic valve and A2 and P2 scallops of the mitral valve (thick arrows)**. The ascending aorta (AA) can also be assessed. (B) Color Doppler is applied to the aortic valve and left ventricular outflow tract.

After completing data acquisition at a 165° imaging plane, the imaging plane is decreased to 135°. *The aortic valve then becomes the focus for orientation of all subsequent views*. The probe is turned to demonstrate the left ventricle long-axis view (Figure 8) with the basal and mid segments of the inferolateral wall and anteroseptum. The right coronary cusp and either the left or non-coronary cusp of the aortic valve as well as the P2 and A2 scallops of the mitral valve are also seen. The aortic and mitral valves are assessed with color Doppler. The probe can be withdrawn slightly to assess the ascending aorta for atherosclerotic plaque and proximal dissection.

**Figure 9:**
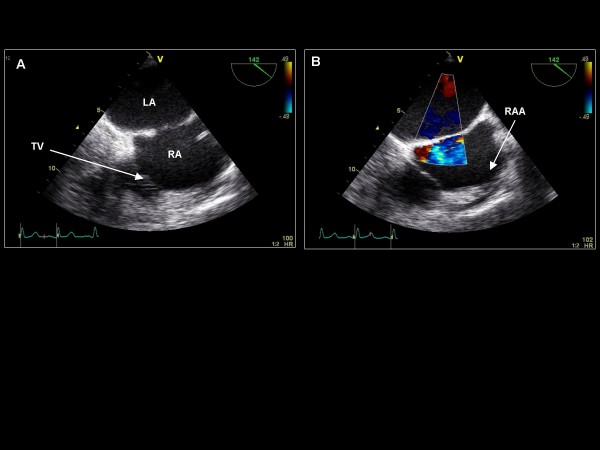
**Imaging plane 135°, (A) The left atrium (LA), interatrial septum, right atrium (RA), and tricuspid valve (TV) are seen (B) The right atrial appendage (RAA) is identified and color Doppler is applied over the interatrial septum demonstrating no interatrial flow**.

Maintaining the imaging angle at 135°, the probe is turned clockwise to demonstrate the right and left atria and interatrial septum (Figure 9). The interatrial septum appears horizontally across the screen, and the fossa ovalis is well visualized. Color Doppler is applied to identify atrial septal defects. This view is helpful for assessing tricuspid valve pathology, the coronary sinus, and pacing wires/right heart catheters. Additionally, the right atrial appendage (RAA) is visualized and pulse wave Doppler is applied to assess emptying velocities. Agitated saline injections for patent foramen ovale detection may be performed at this or other orientations (see later).

**Figure 10:**
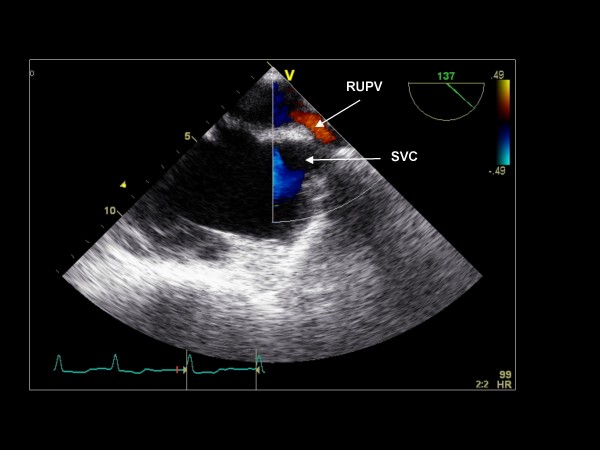
**Imaging plane 135°**. The right upper pulmonary vein (RUPV) and superior vena cava (SVC) are demonstrated.

Maintaining the imaging plane at 135°, further clockwise turning of the probe allows for visualization of the superior vena cava and right upper pulmonary vein (Figure 10). Color Doppler and pulse Doppler of the pulmonary vein are acquired.

### 90° Imaging plane

**Figure 11:**
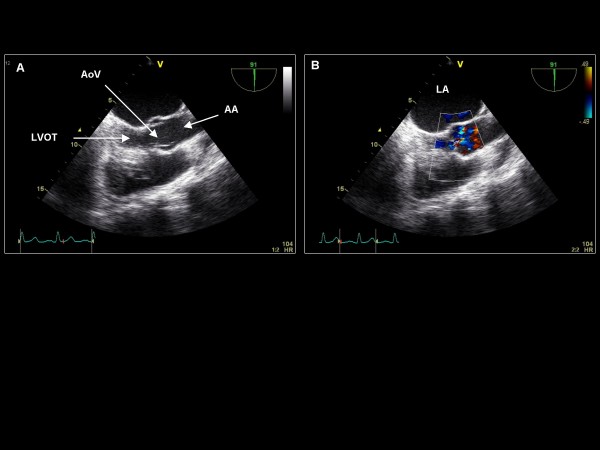
**Imaging plane 90°, (A) The LVOT, aortic valve (AoV), and ascending aorta (AA)**. (B) Color Doppler is applied to the LVOT and aortic valve.

**Figure 12:**
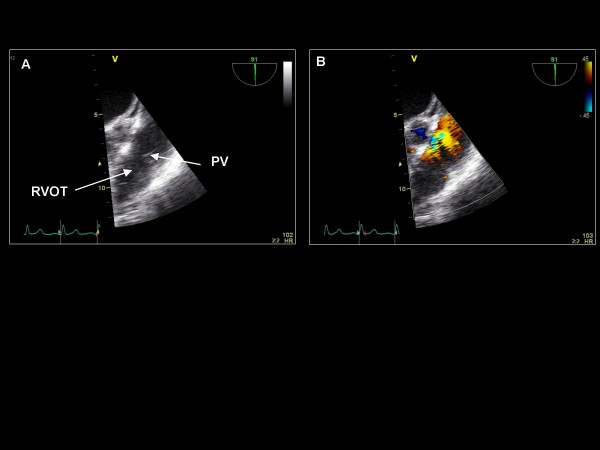
**Imaging plane 90°, (A) The right ventricular outflow tract (RVOT) and pulmonic valve (PV)**. (B) Color Doppler is applied to the pulmonic valve demonstrating trivial pulmonic regurgitation.

**Figure 13:**
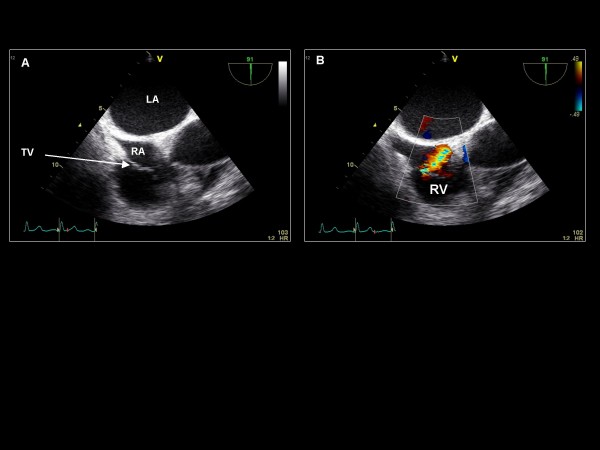
**Imaging plane 90°, (A) The TV, RA, and interatrial septum are visualized**. (B) Color Doppler is applied to the tricuspid valve demonstrating mild tricuspid regurgitation.

**Figure 14:**
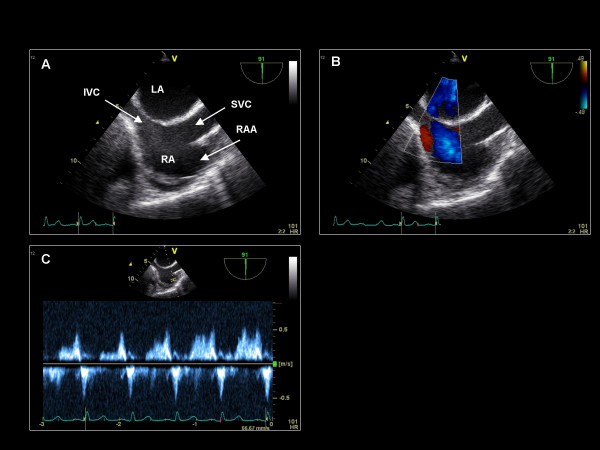
**Imaging plane 90°, (A) The bicaval view demonstrating the SVC, inferior vena cava (IVC), and RAA, and interatrial septum**. (B) Color Doppler applied to the interatrial septum. (C) Pulse wave Doppler of the RAA demonstrating normal emptying velocities in a patient in sinus rhythm.

*Maintaining the imaging plane at 135°, the probe is then turned counterclockwise to view the aortic valve*. The imaging plane is then decreased to 90° for a 2D and color Doppler assessment of the long axis of the left ventricle, the aortic valve and ascending aorta (Figure 11). Slight clockwise turning will demonstrate the right ventricular outflow tract (RVOT) and pulmonic valve (Figure 12). Further clockwise turning will demonstrate the tricuspid valve (Figure 13) where color Doppler is applied as well as CW Doppler (if aligned with flow). Further clockwise turning will demonstrate the bicaval view (Figure 14). In the bicaval view, the interatrial septum traverses horizontally across the screen, and an atrial septal defect can be appreciated with color Doppler. We typically choose this view for agitated saline injection (rest, post-Valsalva, and cough) for a suspected patent foramen ovale, but the previously described 0° (Figure 4) and 135° orientations (Figure 9) or the subsequent 60° orientation (**see later**) may also be used. The RA and RAA are visualized. Pulse wave Doppler of the RAA is performed to assess emptying velocities.

**Figure 15:**
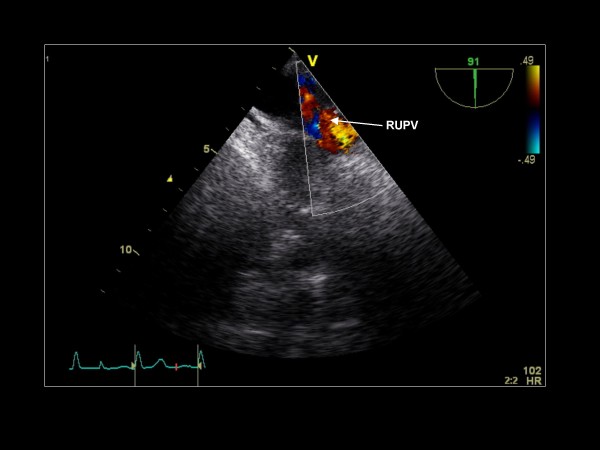
**Imaging plane 90°, The right upper pulmonary vein (RUPV) is well visualized with color Doppler**.

Further clockwise turning of the probe identifies the right upper pulmonary vein (Figure 15). Slight probe advancement and additional clockwise rotation can demonstrate the right lower pulmonary vein. Each pulmonary vein is assessed with color and pulse wave Doppler.

**Figure 16:**
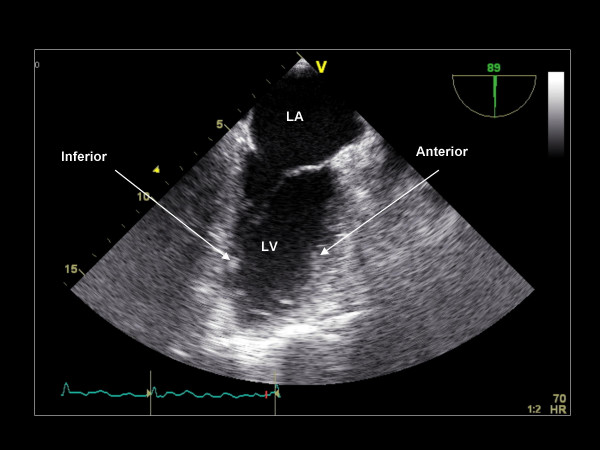
**Imaging plane 90°, The two-chamber view demonstrating the anterior and inferior walls of the left ventricle (LV)**.

**Figure 17:**
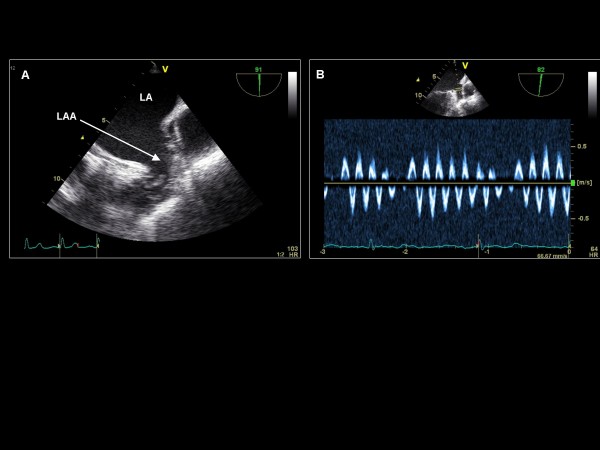
**Imaging plane 90°, (A) The LAA is identified**. (B) Pulse wave Doppler applied to the LAA demonstrating emptying velocities in a patient with atrial flutter.

**Figure 18:**
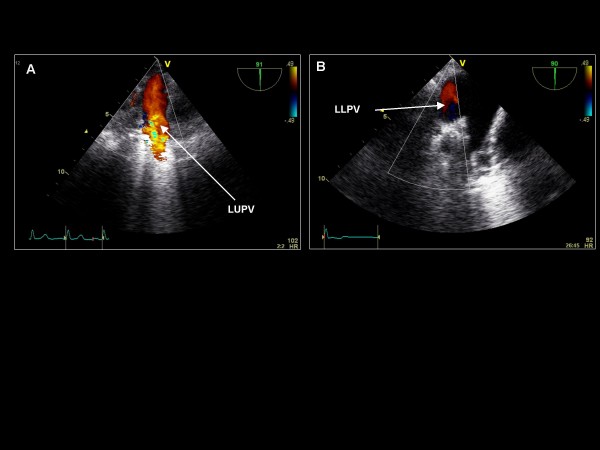
**Imaging plane 90°, (A) Color Doppler demonstration of the left upper pulmonary vein (LUPV) and with further posterior probe rotation (B) the left lower pulmonary vein (LLPV)**.

While maintaining the imaging plane at 90°, the image depth is increased and the TEE probe is turned counterclockwise to obtain the two-chamber view, including the LA, the LV, and the mitral valve (Figure 16). The probe is then withdrawn slightly, anteflexed, and turned more counterclockwise to visualize the LAA at 90° (Figure 17). Pulse wave Doppler is applied to assess emptying velocities. Further counterclockwise turning of the probe brings into view the left upper pulmonary vein and subsequently the left lower pulmonary vein (Figure 18). Each pulmonary vein is assessed with color Doppler and pulse wave Doppler.

### 40 – 60° Imaging plane

**Figure 19:**
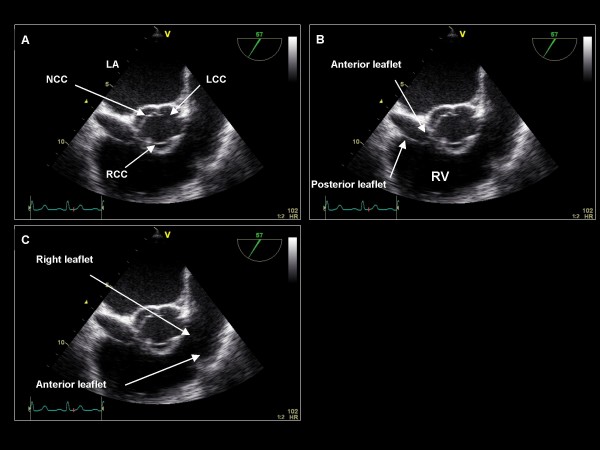
**Imaging plane 50–60°, (A) The short axis view of the aortic valve demonstrating the three coronary leaflets**. (B) The anterior and posterior leaflets of the tricuspid valve. (C) The right and anterior leaflets of the pulmonic valve. NCC = noncoronary cusp, LCC = left coronary cusp, RCC = right coronary cusp.

**Figure 20:**
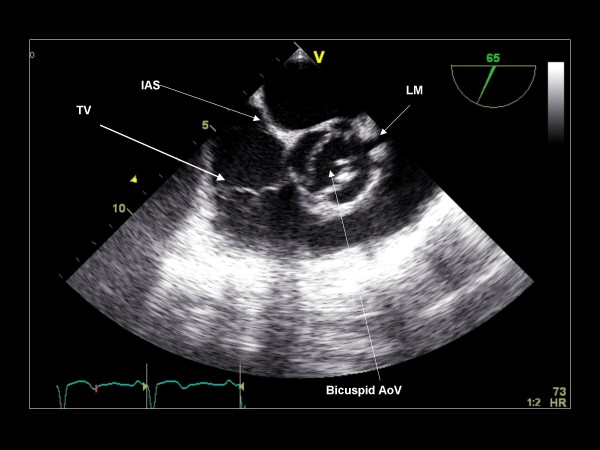
**Imaging plane 65°, The short axis view demonstrating the IAS, TV and a bicuspid aortic valve**. The left main (LM) can be seen in most patients.

*Maintaining the imaging plane at 90°, the probe is then turned clockwise to view the aortic valve*. The image plane is then decreased to 50° – 60°, to obtain the short axis view of the aortic valve (Figure 19). The probe is advanced and withdrawn in this position to assess the anatomy directly below (LVOT) and above the aortic valve. Color Doppler is applied at the aortic valve and LVOT levels to assess for regurgitation. The anterior and posterior leaflets of the tricuspid valve are assessed with and without color Doppler. The right and anterior cusps of the pulmonic valve and main pulmonary artery are also seen in this view [14]. Color Doppler is applied to the pulmonic valve to assess for regurgitation. Clockwise turning of the TEE probe will demonstrate the interatrial septum again. Applications of color Doppler or agitated saline injections can be used to search for an atrial septal defect or patent foramen ovale (Figure 20).

### Imaging plane 0° – Passing Through the Gastroesophageal Junction

**Figure 21:**
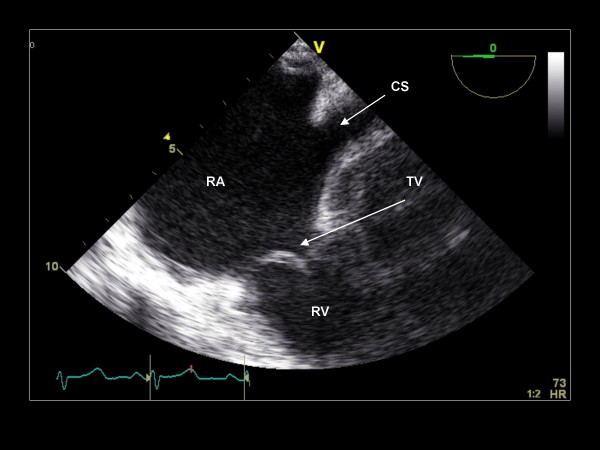
**Imaging plane 0° immediately before passing through the gastroesophageal junction, The RA, RV, TV and coronary sinus (CS) are demonstrated**.

The imaging plane is then decreased to 0° and the mitral valve, LAA, and interatrial septum can be reassessed if further data are desired. Subsequently, the probe is passively advanced through the gastro-esophageal junction as the TEE handle is turned counterclockwise to visualize the coronary sinus (Figure 21).

### Transgastric Views

**Figure 22:**
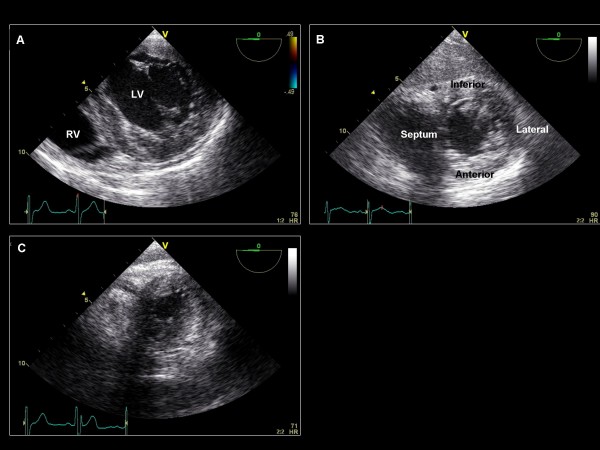
**Transgastric view – imaging plane 0°, short axis view of the LV**. (A) The level of the base demonstrating the mitral valve leaflets. (B) The mid-papillary level. (C) The apical level.

**Figure 23:**
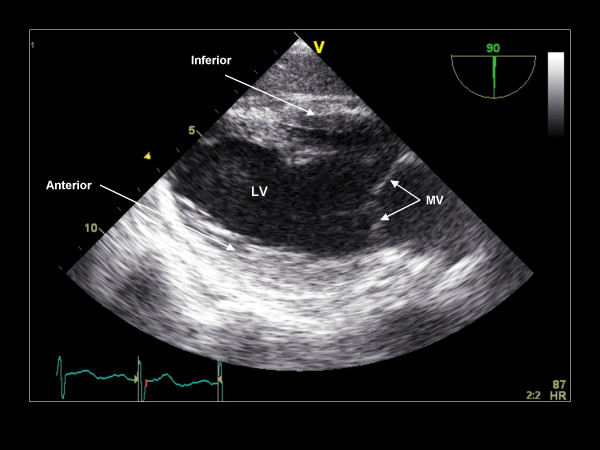
**Imaging plane 90°, The transgastric two-chamber view demonstrating the anterior and inferior LV walls and the mitral valve**.

**Figure 24:**
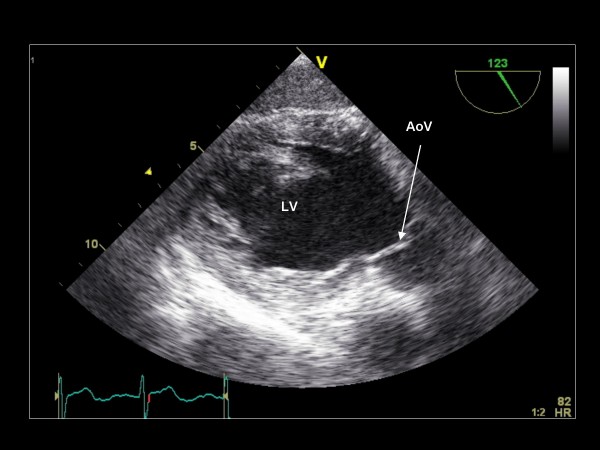
**Imaging plane 120°, The transgastric view of the LV and the aortic valve (AoV)**.

**Figure 25:**
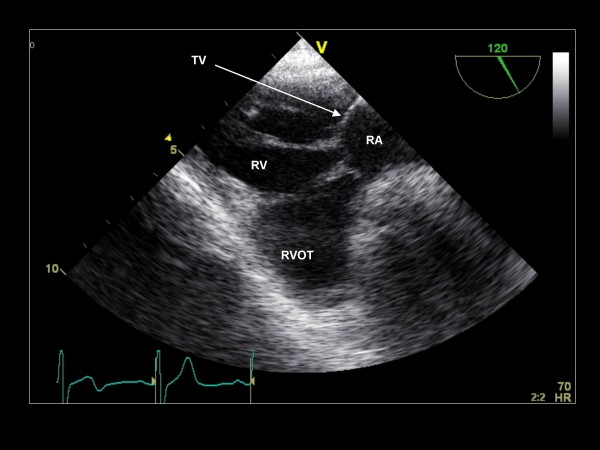
**Imaging plane 120°, The transgastric view of the RA, TV, RV, and right ventricular outflow tract (RVOT)**.

With the imaging plane at 0°, the TEE probe is advanced beyond the gastro-esophageal junction into the stomach to a distance of ~45 cm from the incisors. Once in the stomach, the probe is anteflexed to visualize the short axis of the ventricles (Figure 22). The distance that the TEE probe is advanced determines whether the short axis image is obtained at the level of the mitral valve, mid-ventricle, or apex. With the short axis of the LV centered in the screen, the imaging plane is increased to 90° and the control handle is turned slightly counterclockwise. This provides a 2-chamber view of the left ventricle (Figure 23). The imaging plane is then increased to 120° and the probe is turned clockwise and flexed to demonstrate the left ventricular outflow tract and aortic valve (Figure 24). Color Doppler can be applied to assess for aortic regurgitation and continuous wave (CW) Doppler may be applied to measure the aortic valve gradient. Finally, while maintaining the imaging plane at 120°, the probe is turned clockwise to visualize the right ventricle, right atrium, tricuspid valve, and RVOT with and without color Doppler (Figure 25).

### Thoracic Aorta and Pulmonary Artery

**Figure 26:**
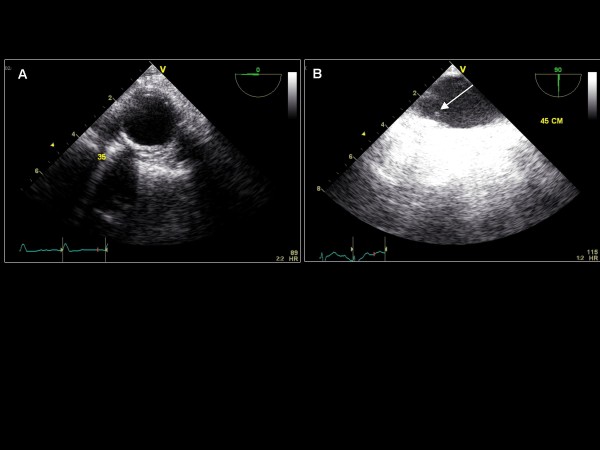
**The thoracic aorta**. (A) 0°, short axis view. (B) 90°, long axis view demonstrating atherosclerotic plaque (arrow). We always label the position of each image (e.g. 45 cm) as the distance from the incisors.

**Figure 27:**
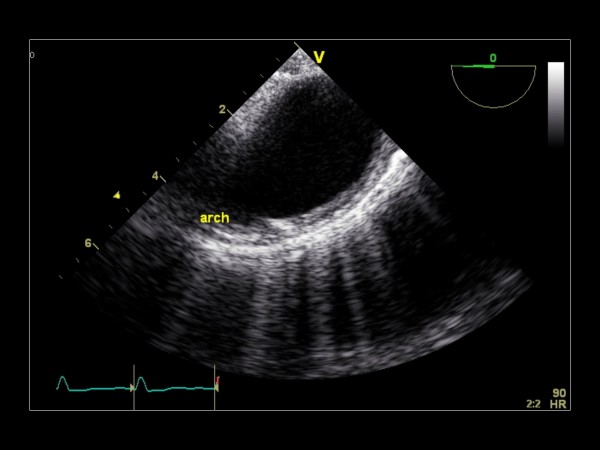
**Imaging plane 0°, The aortic arch**.

**Figure 28:**
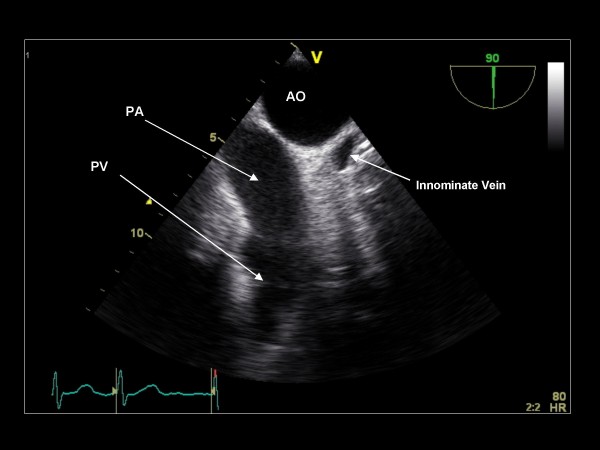
**Imaging plane 90°, The aortic arch (AO), main pulmonary artery (PA), the pulmonic valve (PV), and innominate vein**.

The imaging plane is decreased to 0° and the probe is rotated counterclockwise to demonstrate the thoracic aorta in cross section (Figure 26). The probe is advanced until the aorta is no longer visualized (approximately 44 – 50 cm from the patient's incisors) and is then slowly withdrawn as the aorta is inspected for evidence of atherosclerotic plaque and dissection. To further survey plaque and other vessel wall changes, the imaging plane can be increased to 90° to visualize the long axis of the aorta. As the probe is withdrawn to the upper esophagus, the aortic arch is inspected (Figure 27). The probe is turned counterclockwise and withdrawn further to better visualize the distal ascending aorta. At the level of the arch, the imaging plane is increased to 90°. The pulmonic valve and main pulmonary artery are visualized (Figure 28). Color Doppler and CW Doppler across the pulmonic valve can be applied. The probe is then left in the neutral position and withdrawn from the patient.

### Saline Contrast

Agitated saline contrast is often performed during TEE in patients with suspected paradoxical embolism due to a patent foramen ovale [15]. For these patients, we usually interrogate the interatrial septum from 0–180° to identify the best orientation to visualize the thinnest portion of the interatrial septum/fossa ovalis. The 90° orientation is often best, but this can vary. Saline is administered at rest, followed by injections with cough and post-Valsalva release. Except for cases of suspected persistence of a left sided superior vena cava, our preference is to inject agitated saline from the right arm so as to avoid potential obstruction to venous flow related to the patient's left lateral decubitus position.

## Discussion

In this manuscript, we present an organized guide to the performance of a comprehensive TEE examinationThough not intended to be exclusive, the examination outlined in this manuscript is functionally efficient for both the operator and subsequent image review. Our presentation is distinct from other reports that have described the structures seen in various views/esophageal positions [4-6,11,14] but without an organized approach to performing the TEE.

While not considered exclusive, we hope our approach provides for a more comprehensive assessment of structures related to the specific TEE indication while also providing structure/organization to the image acquisition so as to reduce the likelihood of accidentally omitting any views, which is important for a moderately invasive procedure in which it would be strongly desireable to not to have to repeat the procedure. While we recognize that a focused or abbreviated protocol may be preferred in specific situations (hemodynamic instability; follow-up of atrial appendage thrombus), we believe that a comprehensive TEE should be performed on the vast majority of patients so as to provide a complete assessment of all of the structures of the heart as well as specific views of the thoracic aorta and main pulmonary artery. Using the approach described here, an organized and comprehensive TEE examination can be completed in 10 – 15 minutes.

## Conclusion

An organized guide to the performance of a comprehensive TEE examination is advocated and presented. Such an approach will likely reduce the likelihood of inadvertently omitting a view/structure and also assist in image review.

## Abbreviations

CW: continuous wave; LA: left atrium; LAA: left atrial appendage; LUPV: left upper pulmonary vein; LV: left ventricular; LVOT: left ventricular outflow tract; RA: right atrial; RAA: right atrial appendage; RV: right ventricular; TEE: transesophageal echocardiography; TR: tricuspid regurgitation; TTE: transthoracic echocardiography; TV: tricuspid valve.

## Competing interests

The authors declare that they have no competing interests.

## Authors' contributions

AAK participated in study conception, initial manuscript preparation, manuscript editing and final approval. SBY participated in manuscript editing and final approval. WJM participated in study conception, manuscript editing and final approval.

## Pre-publication history

The pre-publication history for this paper can be accessed here:


